# Hierarchical semantic interaction-based deep hashing network for cross-modal retrieval

**DOI:** 10.7717/peerj-cs.552

**Published:** 2021-05-25

**Authors:** Shubai Chen, Song Wu, Li Wang

**Affiliations:** 1 College of Computer and Information Science, Southwest University, Chongqing, People’s Republic of China; 2 College of Electronic and Information Engineering, Southwest University, Chongqing, People’s Republic of China

**Keywords:** Bidirectional Bi-linear Interaction, Dual-Similarity Measurement, Cross-Modal Hashing, Deep Neural Network

## Abstract

Due to the high efficiency of hashing technology and the high abstraction of deep networks, deep hashing has achieved appealing effectiveness and efficiency for large-scale cross-modal retrieval. However, how to efficiently measure the similarity of fine-grained multi-labels for multi-modal data and thoroughly explore the intermediate layers specific information of networks are still two challenges for high-performance cross-modal hashing retrieval. Thus, in this paper, we propose a novel Hierarchical Semantic Interaction-based Deep Hashing Network (HSIDHN) for large-scale cross-modal retrieval. In the proposed HSIDHN, the multi-scale and fusion operations are first applied to each layer of the network. A Bidirectional Bi-linear Interaction (BBI) policy is then designed to achieve the hierarchical semantic interaction among different layers, such that the capability of hash representations can be enhanced. Moreover, a dual-similarity measurement (“hard” similarity and “soft” similarity) is designed to calculate the semantic similarity of different modality data, aiming to better preserve the semantic correlation of multi-labels. Extensive experiment results on two large-scale public datasets have shown that the performance of our HSIDHN is competitive to state-of-the-art deep cross-modal hashing methods.

## Introduction

The recent exponential growth of multimedia data (e.g., images, videos, audios, and texts) increases the interest in these different modality data. These different modality data, also named multi-modal data, may share similar semantic content or topics. Therefore, cross-modal retrieval, which uses a query from one modality to retrieve all semantically relevant data from another modality, has attracted increasing attention. Because of the existing potential heterogeneous gaps among these multi-modal data, which may be inconsistent in different spaces, it posed a challenge to efficiently and effectively retrieve the related data among these multi-modal data.

Specifically, cross-modal retrieval aims to learn common latent representations for different modalities data so that the embedding of different modalities could be evaluated in the trained latent space (Kaur, Pannu & Malhi, 2021). Many cross-modal retrieval methods are based on real-valued latent representations for modality-irrelevance data, such as Wang et al. (2014), Jia, Salzmann & Darrell (2011), Mao et al. (2013), Gong et al. (2014), Karpathy, Joulin & Fei-Fei (2014), and Wang et al. (2015). However, the measure of real-valued latent representations suffers from the low efficiency of searching and high complexity of computing. To reduce the search time and the storage cost of cross-modal retrieval, hashing-based cross-modal (CMH) retrieval methods are proposed to map the data into compact and modality-specific hash codes in a Hamming space, which have shown their superiority in cross-modal retrieval task such as Ling et al. (2019); Qin (2020).

So far, plentiful CMH algorithms including unsupervised, supervised, and semi-supervised learning manners have been proposed to learn robust hash functions as well as high-quality hash representations. Unsupervised CMH algorithms explore underlying correlation and model the inter and intra-modality similarity among the unlabeled data. In contrast, both semi-supervised and supervised methods employ supervised information, e.g., labels/tags, to learn hash function and hash binary codes, which have better performance than unsupervised manner. However, these CMH algorithms heavily depend on the shallow framework, where the features extraction and hash code projection are two separate steps. Thus, it may limit the robustness of the final learned hash functions and hash representations.

With the remarkable development in the field of artificial neural networks (ANN), deep neural networks (DNN) has shown their high performance at various multimedia tasks, such as Han, Laga & Bennamoun (2019), Girshick (2015), Wu et al. (2017b, 2017a), Guo et al. (2016a, 2016b), Mohammad, Muhammad & Shaikh (2019), Swarna et al. (2020), Muhammad et al. (2021), and Sarkar et al. (2021). Because of the significant capability of DNN in fitting non-linear correlations, it has been widely utilized for the task of cross-modal hashing retrieval, which simultaneously learns robust hash functions and hash representations in an end-to-end deep architecture. Moreover, DNN based models have illustrated great advantages over other hand-crafted shallow models. To name a few, Deep Cross-Modal Hashing (DCMH) (Jiang & Li, 2017), Self-Super Adversarial Hashing (SSAH) Li et al. (2018), Correlation Hashing Network (CHN) (Cao et al., 2016), Self-Constraint and Attention-based Hashing Network (SCAHN) (Wang et al., 2020a), Triplet-based Deep Hashing (TDH) (Deng et al., 2018), Self-Constraining and Attention-based Hashing Network (SCAHN) (Wang et al., 2020b), Pairwise Relationship Guided Deep Hashing (PRDH) (Yang et al., 2017) and Multi-Label Semantics Preserving Hashing (MLSPH) (Zou et al., 2021). However, these DNN based models still suffer from the following disadvantages. Firstly, the single-class label-based supervised information is adopted to measure the semantic similarity between inter and intra-modality instances. However, this oversimple measurement cannot fully exploit the fine-grained relevance, as the pairwise data from inter and intra-modality may share more than one label. Secondly, the abstract semantic features produced by the top layer of DNN are adopted to represent the semantic information of different modalities. However, the representations from the intermediate layer, which has specific information, are neglected. Moreover, this manner cannot fully make use of the multi-scale local and global representations, resulting in suboptimal hash representations.

**Figure 1:**
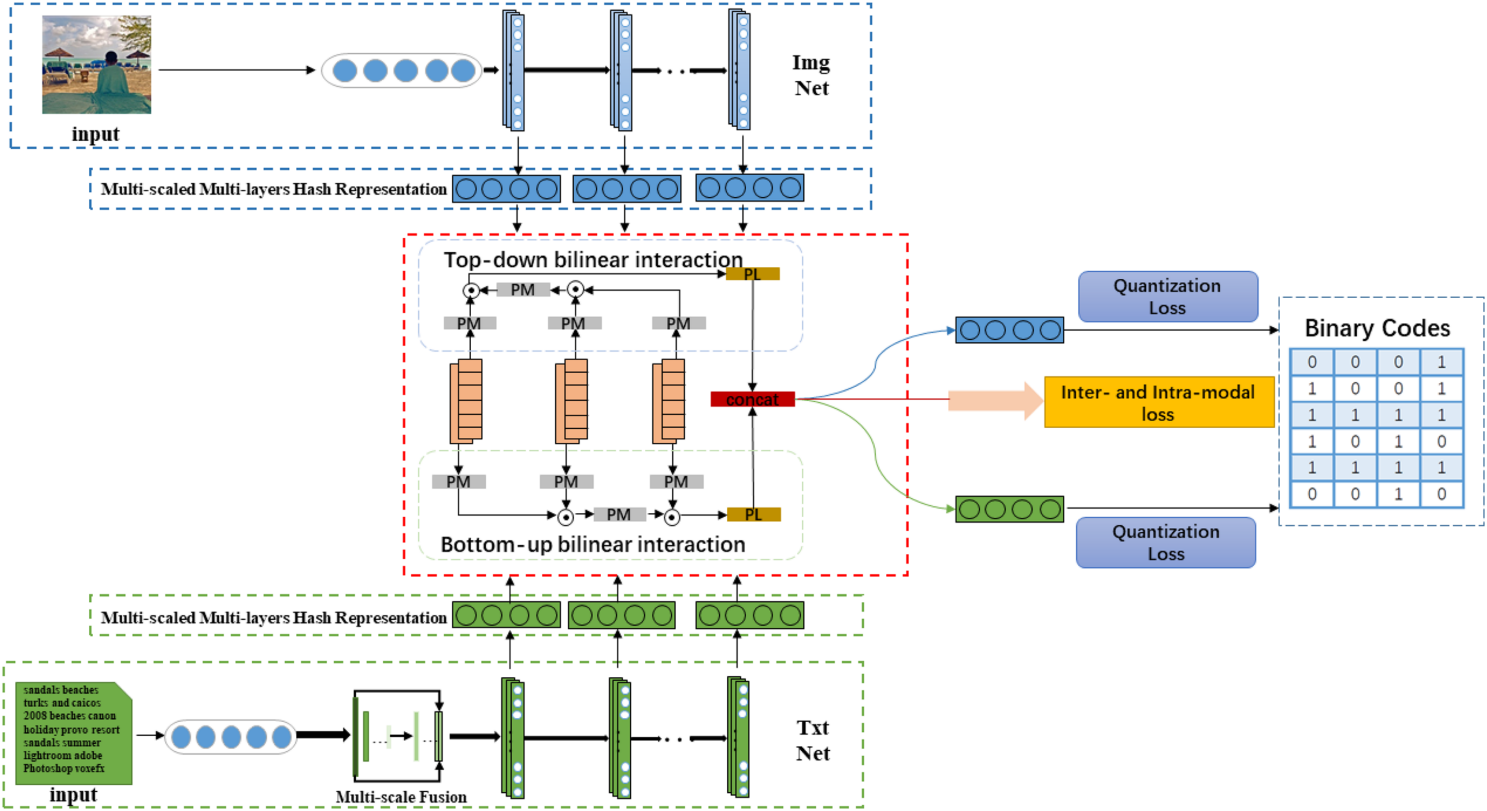
The architecture of our proposed HSIDHN which consists of two parts. One component is the backbone network used to extract hash representations. The other one is the Bidirectional Bi-linear Interaction (BBI) module used to capture the hierarchical semantic correlation of each modality data from different levels.

In this paper, we propose a novel Hierarchical Semantic Interaction-based Deep Hashing Network (HSIDHN) to address the above-mentioned problems. As demonstrated in Fig. 1, the proposed HSIDHN consists of two essential components. One component is the backbone network used to extract the hierarchical hash representations from different modality data (e.g., images and text). The other one is the Bidirectional Bi-linear Interaction (BBI) module used to capture the hierarchical semantic correlation of each modality data from a different level. In the bidirectional bi-linear interaction module, a multi-scale and fusion process is first operated on each layer of the backbone network. The bidirectional interaction policy consisting of a bottom-top interaction and a top-bottom interaction is then designed to exploit the specific semantic information from different layers. And finally, each interaction is aggregated by a bi-linear pooling operation, and the interactions between bottom-up and up-bottom are concatenated together to enhance the capability of hash representations. Moreover, a dual-similarity measurement (“hard” similarity and “soft” similarity) is designed to calculate the semantic similarity of different modality data, aiming to better preserve the semantic correlation of multi-labels. The “hard” similarity means the instances share at least one label, while the “soft” similarity means the distribution difference between two label vectors measured by Maximum Mean Discrepancy (MMD).

The main contributions of HSIDHN are summarized as follows:

- Firstly, a novel bidirectional bi-linear interaction module is designed to achieve hierarchical semantic interaction for different modality data. The bidirectional bi-linear interaction policy could effectively aggregate the hash representations from multiple layers and explore pairwise semantic correlation, promoting significant parts from different layers in a macro view. Therefore, it could enhance the discrimination of final hash representations.
- Secondly, a dual-similarity measurement using both a single class label constraint and Maximum Mean Discrepancy is proposed to map label vectors into the Reproducing Kernel Hilbert Space (RKHS). Thus, the semantic relationship of different modalities, especially instances with multi-labels, can be thoroughly explored.
- Thirdly, we apply the HSIDHN model on two large-scale benchmark datasets with images and text modalities. The experiment results illustrated the HSIDHN surpasses other baseline models on the task of hashing-based cross-modal retrieval.

The rest of this paper is organized as follows. The related work is summarized in “Related Work”. The detailed description of HSIDHN for cross-modal retrieval is presented in “Proposed HSIDHN”. The experimental results and evaluations are illustrated in “Experiment”. Finally, we conclude this paper in “Conclusion”.

## Related Work

### Deep cross-modal hashing

In these years, deep learning has been widely used in cross-modal retrieval tasks due to its appealing performance in various computer vision applications such as Vasan et al. (2020), Dwivedi et al. (2021), Bhattacharya et al. (2020), Gadekallu et al. (2020), Jalil Piran et al. (2020), Jalil Piran, Islam & Suh (2018), and Joshi et al. (2018). It obtains hash representations and hash function learning in an optimal end-to-end framework which also demonstrates the robustness. One of the most typical is deep cross-modal hashing (DCMH) (Jiang & Li, 2017), which firstly applies the deep learning architecture to cross-modal hashing retrieval. The self-constraint and attention-based hashing network (SCAHN) (Wang et al., 2020a) explores the hash representations of intermediate layers in an adaptive attention matrix. The correlation hashing network (CHN) (Cao et al., 2016) adopts the triplet loss measured by cosine distance to reveal the semantic relationship between instances and acquires high-ranking hash codes. Pairwise relationship guided deep hashing (PRDH) (Yang et al., 2017) leverages pairwise instances as input for each modality where supervised information is fully explored to measure the distance of intra- and inter-modality, respectively. Cross-modal hamming hashing (CMHH) (Cao et al., 2018) learns high-quality hash representations and hash codes with a well-designed focal loss and a quantization loss. Although these algorithms mentioned above have obtained high performance in CMH tasks, they ignore the rich spatial information from intermediate layers, which is essential to the modality-invariant hash representations learning procedure.

### Multi-label similarity learning

In the real-world scenario or benchmark datasets, instances are always related to multiple labels. Thus multi-label learning has attracted more and more attention in various applications. However, most existing CMH methods adopt single label constraints to measure the similarity among intra- and inter-modality instances. Self-supervised adversarial hashing (SSAH) (Li et al., 2018), which uses an independent network to learn multi-label representations, and thus the semantic correlations are preserved. However, it only takes the multi-label information to supervise the label network training, and the original images or text are still measured by single-label. Improved deep hashing network (IDHN) (Zhang et al., 2019) introduces pairwise similarity metrics to fit the multi-label instances applications. In contrast, this method concentrates on the single modality hashing retrieval. Different from these methods that apply multi-label information, our HSIDHN employs both single-label and multi-label constraint to learn more robust hash representations. Significantly, the Maximum Mean Discrepancy (MMD) is adopted as the multi-label calculation criterion. To our knowledge, the HSIDHN is the first method using MMD in the deep CMH framework.

## Proposed HSIDHN

In this section, the problem definition, the details of Hierarchical Semantic Interaction-based Deep Hashing Network (HSIDHN), including feature extraction architecture, are presented one by one. Without losing generality, we assume each instance has both image-modality and text-modality. However, it can be easily extended to other modalities such as videos, audios and graphics.

### Problem definition

We use uppercase letters to represent matrices, such as *X*, and lowercase letters representing vectors, such as *y*. The transpose of *G* are denoted as *G*^*T*^, and sign function sign(·) is defined as:

(1) $$\begin{array}{c} sign(x)=\{\begin{array}{cc} 1 & x\geq 0\\ -1 & x<0 \end{array} \end{array}$$

We assume the training dataset $O={\left\{o_{i}\right\}}_{i=1}^{N}$ with *N* instances, which all of them have label information and image-text modality feature vectors. The *ith* training instance is denoted as $o_{i}=\left(v_{i},t_{i}\right)$, where *v*_*i*_ ∈ $R^{d_{v}}$ and *t*_*i*_ ∈ $R^{d_{t}}$ denote the *d*_*v*_ and *d*_*t*_ dimensional feature vectors of image and text respectively. Moreover, the label based semantic similarity matrix is defined as $S_{*}=\left\{S_{*}^{vt},S_{*}^{vv},S_{*}^{tt}\right\}$, where $S_{*}^{vv}=\left\{S_{ij}^{vv}|i,j=1,2,\ldots ,N\right\}\in R^{N\times N}$ and $S_{*}^{tt}=\left\{S_{ij}^{tt}|i,j=1,2,\ldots ,N\right\}\in R^{N\times N}$ denote the intra-modality similarity matrix of image and text, $S_{*}^{vt}=\left\{S_{ij}^{vt}|i,j=1,2,\ldots ,N\right\}\in R^{N\times N}$ denotes the inter-modality similarity matrix between image and text. S_*_ means the “hard” similarity and “soft” similarity when * = *h* or * = *r*.

Given the training datasets *O* and *S*, the main objective of our proposed HSIDHN is to learn two modality discriminative hash functions *h*^(*v*)^(**v**) and *h*^(*t*)^(**t**) for image and text modalities, which can map features vectors into a compact binary space and preserve relationship and correlation among instances. The learning framework can be roughly divided into two parts, hash representations learning section and hash function learning section. Therefore, $F=\left\{f_{v_{i}}|i=1,2,\cdots ,N\right\}\in R^{N\times c}$ and $G=\left\{g_{t_{i}}|i=1,2,\cdots ,N\right\}\in R^{N\times c}$ are used to denote the learned hash representations of image-modality and text-modality. Besides, $B=\left\{B_{i}|i=1,2,\cdots ,N\right\}\in R^{N\times c}$ is the projection of the final hash codes from *F* and *G* by simply using a sign function *B* = sign(*F* + *G*).

The architecture of our proposed HSIDHN which consists of two parts. One component is the backbone network used to extract hash representations. The other one is the Bidirectional Bi-linear Interaction (BBI) module used to capture the hierarchical semantic correlation of each modality data from different levels.

### Network framework of HSIDHN

For most cross-modal hashing retrieval methods, the multi-level and multi-scale information cannot be fully explored. Thus, it may limit the invariance and discrimination of the final learned hash representations. In this paper, we propose a novel Hierarchical Semantic Interaction-based Deep Hashing Network (HSIDHN) for large-scale cross-modal retrieval, where a multi-level and multi-scale interaction based network and bidirectional bi-linear interaction module are used to explicitly specifics spatial and semantic information. The general architecture of our proposed HSIDHN is shown in Fig. 1.

**Figure 2:**
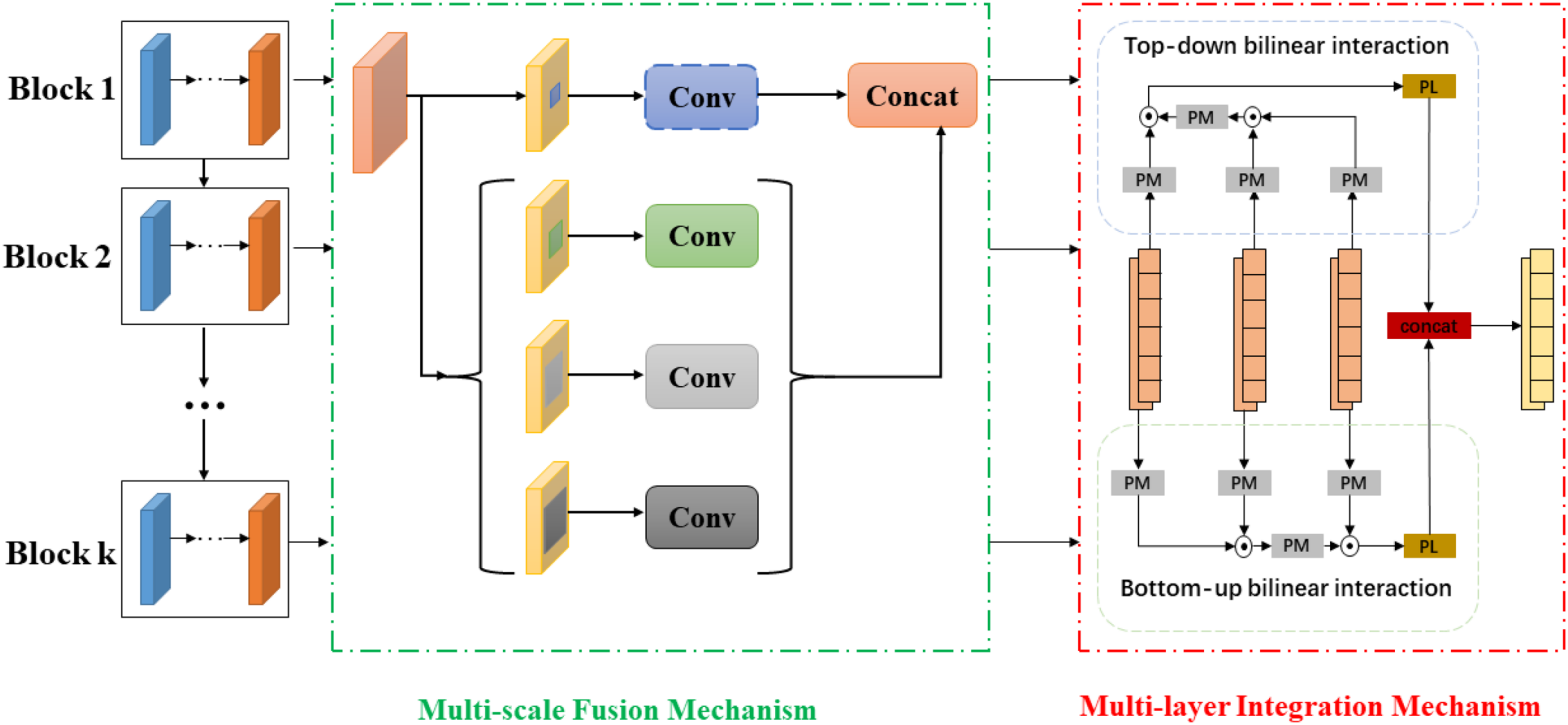
The generation of multi-scale and multi-level hash representations.

In terms of the multi-level and multi-scale hash representations generation, HSIDHN contains double end-to-end network to learn hash functions and hash representations from text and image modality. The deep feature extraction procedure is conducted on Resnet (He et al., 2016), and pair-wise pairs of images and text are applied as input for the Image Network and Text Network. For the Text Network, the bag-of-words (BoW) vector policy has been widely adopted to extract features from Text Networks since Jiang & Li (2017). However, it is inappropriate to learn rich features demanded by the hash functions learning procedure because of BoW vectors’ sparsity. To solve this issue, a multi-scale operation is leveraged by multiple pooling layers, and the vectors are resized by bi-linear-interpolation. Finally, these vectors are concatenated together and fed to the text network, which consequently is helpful to construct semantic correlation for the text. Both image and text networks generate multi-level feature information from mid-layers by exploring an adaptive average pooling. Motivated by SPPNet (Purkait, Zhao & Zach, 2017), the multi-scale fusion structure is also applied to hash representations from each layer to obtain rich spatial information. Therefore, the semantic relevance and correlation from different layers can be fully explored to enhance the invariance of hash representations for both image and text modality. The whole architecture is shown in Fig. 2.

As different layers of the network have complementary hash representations. Thus the interaction among different layers may help to learn discriminative hash representations. A bidirectional bi-linear integration module integrates the multi-scaled multi-level hash representations from intermediate layers to learn more robust hash representations. The bidirectional bi-linear integration policy has two main procedures, the bottom-up and top-down progress. The bottom-up procedure allows shadow activation, which always being covered by the top layers to accumulate slowly. The top-down operation can take advantage of contextual and spatial information. Therefore, the combination of bottom-up and top-down interaction policy could generate better hash representations. We assume there are *K* multi-scaled and multi-levels hash representations generated from Resnet (He et al., 2016), and the *f*^(*k*)^ and *g*^(*k*)^ are output from *K*^*th*^ block.

Feeding an instance to the network, the output feature map is *X* ∈ *R*^*c*^, where c is the dimension of *X*, and *Z* ∈ *R*^*o*^ is the bi-linear representation with dimension *o*. For *Z*_*i*_ in $z=\left[z_{1},z_{2},\cdots ,z_{o}\right]$, the bi-linear pooling interaction can be defined as:

(2) $$z_{i}=I(x,x)=x^{T}W_{i}x$$

where *W*_*i*_ is the weighted projection matrix need to be learned, *I*(x, x) is the interaction function. According to Rendle (2010), the weighted projection matrix in Eq. (2) can be rewritten by factorizing as:

(3) $$z_{i}=I(x,x)=x^{T}U_{i}V_{i}^{T}x=U_{i}^{T}x\circ V_{i}^{T}x$$

where *U*_*i*_ ∈ *R*^*c*^ and *V*_*i*_ ∈ *R*^*c*^. And consequently, the output features *z* is calculated as:

(4) $$z=P^{T}\left(U^{T}x\circ V^{T}x\right)$$

where *U*, *V* and *P* are projection matrices.

The proposed bidirectional bi-linear integration policy aims to explore the interaction between intermediate layers. Taking the image modality as an example, we firstly select two layers *f*_*i*_ and *f*_*j*_ from multi-scaled multi-level hash representations. Hence, inspired by Yu et al. (2018), the Eq. (4) can be rewritten as:

(5) $$z=P^{T}\left(U^{T}f_{i}\circ V^{T}f_{j}\right)$$

where *P*,*U*,*V* are projection matrices. To reduce number of parameters, the bi-linear pooling is divided into two stages, which can be formulated as:

(6) $$PB=U^{T}f_{i}\circ V^{T}f_{j}$$

(7) $$PL=P^{T}PB$$

Thus, the interaction between two layers can be defined as:

(8) $$Z==P^{T}pool\left(PB\left(X_{1}\right)\circ PB\left(X_{2}\right)\right)$$

(9) $$=P^{T}pool\left(I\left(X_{1},X_{2}\right)\right)$$

In this paper, the interaction is applied on multi-layer and the representation of each layers is defined as:

(10) $$Z_{v}=BI\left(f_{1},f_{2},f_{3}\right)=P^{T}concat\left[I\left(f_{1},f_{2}\right),I\left(f_{1},f_{3}\right),I\left(f_{2},f_{3}\right)\right]$$

(11) $$\begin{array}{c} Z_{t}=BI\left(g_{1},g_{2},g_{3}\right) \end{array}=P^{T}concat\left[I\left(g_{1},g_{2}\right),I\left(g_{1},g_{3}\right),I\left(g_{2},g_{3}\right)\right]$$

where *f*_1_, *f*_2_, *f*_3_ and *g*_1_, *g*_2_, *g*_3_ are hash representations from different layers of image and text modality, and *concat* denotes the concatenation operation. However, the bi-linear pooling operation from one direction may lead to a vanishing gradient problem. This is because parameters from intermediate layers update faster than the end. Thus, the bidirectional bi-linear integration policy can be written as:

(12) $$\begin{array}{l} Z_{v}=BBI\left(f_{1},f_{2},f_{3}\right)=P^{T}\text{concat}\left[Z_{1},Z_{2}\right]\\ =P^{T}\text{concat}[\underset{\text{bottom-top}}{\underset{︸}{I\left(\cdots I\left(I\left(f_{1},f_{2}\right),f_{3}\right)\right)}},\;\underset{\text{top-down}}{\underset{︸}{I\left(I\left(I\left(f_{3},f_{2}\right),f_{1}\right)\cdots ,f_{1}\right)}}], \end{array}$$

(13) $$\begin{array}{l} Z_{t}=BBI\left(g_{1},g_{2},g_{3}\right)=P^{T}\text{concat}\left[Z_{1},Z_{2}\right]\\ =P^{T}\text{concat}[\underset{\text{bottom-top}}{\underset{︸}{I\left(\cdots I\left(I\left(g_{1},g_{2}\right),g_{3}\right)\right)}},\;\underset{\text{top-down}}{\underset{︸}{I\left(I\left(I\left(g_{3},g_{2}\right),g_{1}\right)\cdots ,g_{1}\right)}}], \end{array}$$

where *f*_1_, *f*_2_, *f*_3_ and *g*_1_, *g*_2_, *g*_3_ are hash representations from different layers of image and text modality and *concat* denotes the concatenation operation. And *Z*_1_ and *Z*_2_ are the multi-layer interaction from bottom-up and top-down procedure.

#### Dual-similarity measurement

For most cross-modal retrieval benchmark datasets, it is common for an image or text to have multiple labels. Thus, the traditional methods, which only explore if labels are shared among instances, are not suitable for this situation. Therefore, to enhance the quality of similarity measurement, we propose a Dual-similarity evaluation strategy.

#### “Hard” similarity based hamming distance loss

We use $S_{h}=\left\{S_{h}^{vv},S_{h}^{tt},S_{h}^{vt}\right\}\in \{0,1\}$ to represent the “hard” similarity matrix. In this scenario, the similarity definition follows the identical way which is similar to the previous methods. Given the training instances *o*_*i*_ and *o*_*j*_, the element of similarity matrix $S_{ij_{h}}=1$ means the instances share at least one label, and thus the inner product of these two instance should be large, and $S_{ij_{h}}=0$ otherwise. In the ImgNet and TxtNet, there are *k* parts of features, the *k* − *th* parts of the hash representations from networks are denotes as $f_{v_{i}}^{k}$ and $g_{t_{j}}^{k}$. The likelihood function of image and text inter- and intra-instances are calculated as:

(14) $$p\left(S_{ij_{h}}^{vv}|f_{v_{i}}^{k},f_{v_{j}}^{k}\right)=\{\begin{array}{cc} \sigma \left(\theta_{ij}\right), & S_{ij_{h}}^{vv}=1\\ 1-\sigma \left(\theta_{ij}\right), & S_{ij_{h}}^{vv}=0 \end{array}$$

where $\theta_{ij}=\alpha f_{v_{i}}^{k}f_{v_{j}}^{kT}$.

(15) $$p\left(S_{ij_{h}}^{tt}|g_{t_{i}}^{k},g_{t_{j}}^{k}\right)=\{\begin{array}{cc} \sigma \left(\theta_{ij}\right), & S_{ij_{h}}^{tt}=1\\ 1-\sigma \left(\theta_{ij}\right), & S_{ij_{h}}^{tt}=0 \end{array}$$

where $\theta_{ij}=\alpha g_{t_{i}}^{k}g_{t_{j}}^{kT}$.

(16) $$p\left(S_{ij_{h}}^{vt}|f_{v_{i}}^{k},g_{t_{j}}^{k}\right)=\{\begin{array}{cc} \sigma \left(\theta_{ij}\right), & S_{ij_{h}}^{vt}=1\\ 1-\sigma \left(\theta_{ij}\right), & S_{ij_{h}}^{vt}=0 \end{array}$$

where $\theta_{ij}=\alpha f_{v_{i}}^{k}g_{t_{j}}^{kT}$. *α* is a control hyper-parameter to self-adapt in different length of binary codes, which the value is set to $\alpha =2^{-\log_{2}^{\left(c/64\right)}}$ and $\sigma \left(\theta_{ij}\right)=\frac{1}{1+e^{-\theta_{ij}}}$. The Hamming distance intra-loss of image and text and inter-loss can be defined as:

(17) $$\begin{array}{cc} L_{\text{intra-image}} & =\sum_{k=1}^{K}\left(-\sum_{i,j=1}^{N}\log p\left(S_{ij_{h}}^{vv}|f_{v_{i}}^{k},f_{v_{j}}^{k}\right)\right)\\ & =-\sum_{k=1}^{K}\sum_{i,j=1}^{N}\left(S_{ij_{h}}^{vv}\theta_{v_{i}^{k}v_{j}^{k}}-\log\left(1+e^{\theta_{i}^{k}v_{j}^{k}}\right)\right) \end{array}$$

(18) $$\begin{array}{cc} L_{\text{intra-text}} & =\sum_{k=1}^{K}\left(-\sum_{i,j=1}^{N}\log p\left(S_{ij_{h}}^{tt}|g_{t_{i}}^{k},g_{t_{j}}^{k}\right)\right)\\ & =-\sum_{k=1}^{K}\sum_{i,j=1}^{N}\left(S_{ij_{h}}^{tt}\theta_{t_{i}^{k}t_{j}^{k}}-\log\left(1+e^{\theta_{i}^{k}t_{j}^{k}}\right)\right) \end{array}$$

(19) $$\begin{array}{cc} L_{\text{inter}} & =\sum_{k=1}^{K}\left(-\sum_{i,j=1}^{N}\log p\left(S_{ij_{h}}^{vt}|f_{v_{i}}^{k},g_{t_{j}}^{k}\right)\right)\\ & =-\sum_{k=1}^{K}\sum_{i,j=1}^{N}\left(S_{ij_{h}}^{vt}\theta_{v_{i}^{k}t_{j}^{k}}-\log\left(1+e^{\theta_{v_{i}}t_{j}k}\right)\right) \end{array}$$

The overall “hard” similarity based hamming distance loss can be written as:

(20) $$L_{h}=L_{\text{intra-image}}+L_{\text{intra-text}}+L_{inter}$$

#### “Soft” similarity based mean square error loss

We use $S_{r}=\left\{S_{r}^{vv},S_{r}^{tt},S_{r}^{vt}\right\}\in [0,1]$ to represent the “Soft” Similarity matrix. In this scenario, we use the Maximum Mean Discrepancy (MMD) (Borgwardt et al., 2006) to measure the distance between two label vectors by projecting the original vector into a Reproducing Kernel Hilbert Space (RKHS). The “Soft” Similarity is defined as:

(21) $$\begin{array}{cc} S_{r}^{vt} & =MMD\left(l^{v},l^{t}\right)\\ & ={\left\|\frac{1}{n}\sum_{i=1}^{n}\phi \left(l_{i}^{v}\right)-\frac{1}{n}\sum_{j=1}^{n}\phi \left(l_{j}^{t}\right)\right\|}_{H}^{2} \end{array}$$

where *l*^*v*^ and *l*^*t*^ denotes label vectors of image and text instances.

It is hard to find a suitable projection function *ϕ*(.) in cross-modal retrieval tasks. Thus, the formula of “Soft” is expanded as:

(22) $$\begin{array}{cc} S_{r}^{vt} & =MMD\left(l^{v},l^{t}\right)=∥\frac{1}{n^{2}}\sum_{i}^{n}\sum_{i^{{}^{\prime }}}^{n}\phi \left(l_{i}^{v}\right)\phi \left(l_{i}^{v^{\prime }}\right)\\ & -\frac{2}{nm}\sum_{i}^{n}\sum_{j}^{m}\phi \left(l_{i}^{v}\right)\phi \left(l_{j}^{t}\right)+\frac{1}{m^{2}}\sum_{j}^{m}\sum_{j^{{}^{\prime }}}^{m}\phi \left(l_{j}^{t}\right)\phi \left(l_{j}^{t}\right){∥}_{H}^{2} \end{array}$$

We can easily calculate the above formula by the kernel function *k*(*). The final definition of “Soft” Similarity is shown as:

(23) $$\begin{array}{cc} S_{r}^{vt} & =MMD(l^{v},l^{t})=∥\frac{1}{n^{2}}\sum_{i}^{n}\sum_{i^{{}^{\prime }}}^{n}\phi \left(l_{i}^{v}\right)\phi \left(l_{i}^{v^{\prime }}\right)-\\ & \frac{2}{nm}\sum_{i}^{n}\sum_{j}^{m}\phi \left(l_{i}^{v}\right)\phi \left(l_{j}^{t}\right)+\frac{1}{m^{2}}\sum_{j}^{m}\sum_{j^{{}^{\prime }}}^{m}\phi \left(l_{j}^{t}\right)\phi \left(l_{j}^{t^{\prime }}\right){∥}_{H}^{2} \end{array}$$

where *l*^*v*^ is the label information of image modality and *l*^*t*^ is the label information of text modality. $\left\{i,j\right\}\in R^{1\times n}$ denotes the number of instances.

Thus, according to Eq. (23), we apply this metric to define pairwise intra-modality similarity for image-modality and text-modality as:

(24) $$\begin{array}{cc} S_{r}^{vv} & =MMD(l^{v},l^{v})=∥\frac{1}{n^{2}}\sum_{i}^{n}\sum_{i^{{}^{\prime }}}^{n}\phi \left(l_{i}^{v}\right)\phi \left(l_{i}^{v^{\prime }}\right)-\\ & \frac{2}{nm}\sum_{i}^{n}\sum_{j}^{m}\phi \left(l_{i}^{v}\right)\phi \left(l_{j}^{v}\right)+\frac{1}{m^{2}}\sum_{j}^{m}\sum_{j^{{}^{\prime }}}^{m}\phi \left(l_{j}^{v}\right)\phi \left(l_{j}^{v^{\prime }}\right){∥}_{H}^{2} \end{array}$$

(25) $$\begin{array}{cc} S_{r}^{tt} & =MMD(l^{t},l^{t})=∥\frac{1}{n^{2}}\sum_{i}^{n}\sum_{i^{{}^{\prime }}}^{n}\phi \left(l_{i}^{t}\right)\phi \left(l_{i}^{t^{\prime }}\right)-\\ & \frac{2}{nm}\sum_{i}^{n}\sum_{j}^{m}\phi \left(l_{i}^{t}\right)\phi \left(l_{j}^{t}\right)+\frac{1}{m^{2}}\sum_{j}^{m}\sum_{j^{{}^{\prime }}}^{m}\phi \left(l_{j}^{t}\right)\phi \left(l_{j}^{t^{\prime }}\right){∥}_{H}^{2} \end{array}$$

where *k*(*) is the Gaussian kernel since it can map the original label information to an infinite dimension.

As the similarity is continuous, the Mean Square Error (MSE) loss function is adopted to adapt the “Soft” Similarity. Besides, we apply both inter-and intra-modality constraint loss to bridge the heterogeneous gap and preserve the semantic relevance. Thus, the MSE loss is calculated as:

(26) $$L_{inter_{r}}=\sum_{i=1,j=1}^{n}{\left\|\frac{\left\langle f_{i},g_{j}\right\rangle +c}{2}-s_{ijr}^{vt}\cdot c\right\|}^{2}$$

(27) $$L_{intra-image_{r}}=\sum_{i=1,j=1}^{n}{\left\|\frac{\left\langle f_{i},f_{j}\right\rangle +c}{2}-s_{ijr}^{vv}\cdot c\right\|}^{2}$$

(28) $$L_{intra-text_{r}}=\sum_{i=1,j=1}^{n}{\left\|\frac{\left\langle g_{i},g_{j}\right\rangle +c}{2}-s_{ijr}^{tt}\cdot c\right\|}^{2}$$

where *f*_*i*_ represents the hash representation of *ith* image instance, *g*_*j*_ represents the hash representation of *jth* text instance and *c* is the length of binary codes. Since the inner product ⟨∗,∗⟩∈[−c,c], the value range of $\frac{\left\langle *,*\right\rangle +c}{2}$ will be the same as $s_{ijr}^{**}\cdot c$.

The overall “Soft” Similarity based MSE loss can be written as:

(29) $$L_{r}=L_{inter_{r}}+L_{intra-image_{r}}+L_{intra-text_{r}}$$

#### Quantization loss

The purpose of the dual-similarity based loss function is to guarantee the hash representations *F*, and *G* can preserve similarity. While the similarity of hash codes *B*^(*v*)^ = *sign*(*F*) and *B*^(*t*)^ = sign(*g*) has been neglected. Therefore, we also need to make sure that the binary codes *B*_(*v*)_ and *B*_(*t*)_ preserve the similarity, which is also the goal of cross-modal retrieval. As both *B*_(*v*)_ and *B*_(*t*)_ share the same label information in a mini-batch, the hash codes is set to *B*^(*v*)^ = *B*^(*t*)^ = *B*. Accordingly, the quantization loss is defined as:

(30) $$L_{q}=\frac{1}{c}\left(∥B-F{∥}_{F}^{2}+∥B-G{∥}_{F}^{2}+∥F-G{∥}_{F}^{2}\right)$$

### Optimization

By aggregating the Eqs. (20), (29) and (30), we get the general objective function as:

(31) $$\underset{B,\theta_{x},\theta_{y}}{\min}L=\underset{B,\theta_{x},\theta_{y}}{\min}\left(L_{h}+\gamma L_{r}+\beta L_{q}\right)$$

where *θ*_*x*_ and *θ*_*y*_ are network parameters of image and text, and *B* is the learned binary codes. *γ* and *β* are hyper-parameters to control each part’s weights in the general objective function. We adopt an alternating optimization algorithm, and some parameters are fixed while other parameters are optimized.

### Fix B, optimize θ_*x*_ and θ_*y*_

The back propagation (BP) algorithm is adopted to update parameters *θ*_*Dx*_, *θ*_*Dy*_ by descending gradients:

(32) $$\theta \leftarrow \theta -\eta \cdot \nabla_{\theta }\frac{1}{n}L$$

### Fix θ_*x*_ and θ_*y*_, optimize B

As the *θ*_*x*_ and *θ*_*y*_ is fixed, the optimization of binary codes *B* can be defined as:

(33) $$\begin{array}{l} \underset{B}{\min}tr\left(B^{T}(\eta (F+G))\right)=\eta \sum_{i,j}B_{ij}\left(F_{ij}+G_{ij}\right)\\ \;s.t.\;B\in {\left\{-1,+1\right\}}^{c\times N} \end{array}$$

which can be formulated as:

(34) $$\begin{array}{c} B=sign(\eta (F+G)) \end{array}$$

## Experiment

To evaluate the algorithm we proposed, two large-scale public datasets MIRFlickr-25k (Huiskes & Lew, 2008) and NUS-WIDE (Chua et al., 2009) are employed as our training data to compare with other sate-of-the-art cross-modal hashing methods.

### Datasets

**MIRFlickr-25K (Huiskes & Lew, 2008)**: There are 25,000 instances images in the MIRFlickr-25K collected from Flickr with several textual descriptions. Following the standard experimental settings proposed in DCMH (Jiang & Li, 2017), 20,015 data are samples are leveraged with less than 24 distinct labels. A 1,386-dimensional BoW vector is generated for each text description.

**NUS-WIDE (Chua et al., 2009)**: There are 269,468 image-text instances pair belonging to 81 categories collected from real-world web datasets. Each textual description for image instance is represented by a 1,000-dimensional binary vector. In this paper, 21 of the most frequently used categories are chosen with 190,421 images and related text.

We randomly select 10,000 and 10,500 instances from MIRFlickr-25K and NUS-WIDE as the training set to reduce the computational cost. Meanwhile, we randomly choose 2,000 and 2,100 samples as the query set for MIRFlickr-25k and NUS-WIDE, respectively. The remained data are leveraged as a retrieval set after the query set is selected. Images are normalized before inputting to the network. The details of dataset division are summarized in Table 1.

**Table 1:** Details of datasets division.

Dataset name	Total number	Training set/test set
MIRFLlickr-25K	20,015	10,000/2,000
NUS-WIDE	190,421	10,500/2,100

### Implementation details

Our HSIDHN is implemented using the Pytorch (Paszke et al., 2019) framework and performed on one TITAN Xp GPU server. In the end-to-end framework, Resnet-34 is applied as the backbone network. For the bidirectional bi-linear interaction module, the last three parts of hash representations are integrated to enhance the capability hash representations, respectively. Moreover, the multi-scale fusion of text is applied on pooling sizes of 1, 5, 10, 15, 30. The image network parameters initialization is pre-trained on the ImageNet (Russakovsky et al., 2015) dataset, and the network for text-modality is initialized by Normal distribution $N\left(\mu ,\sigma^{2}\right)$ with *μ* = 0 and *σ* = 0.1. Learning rate is initialized in 10^−1.1^ and gradually decays to 10^−6.1^, and the mini-batch size is 128. Besides, we use the SGD as our optimization for image and text networks.

### Evaluation and baselines

To measure CMH methods’ performance, we adopt hamming ranking as the retrieval protocol, which sorting instances by hamming distance. In this paper, the PR Curves and Mean Average Precision (MAP) (Liu et al., 2014) are leveraged as the evaluation criteria for HSIDHN.

**Figure 3:**
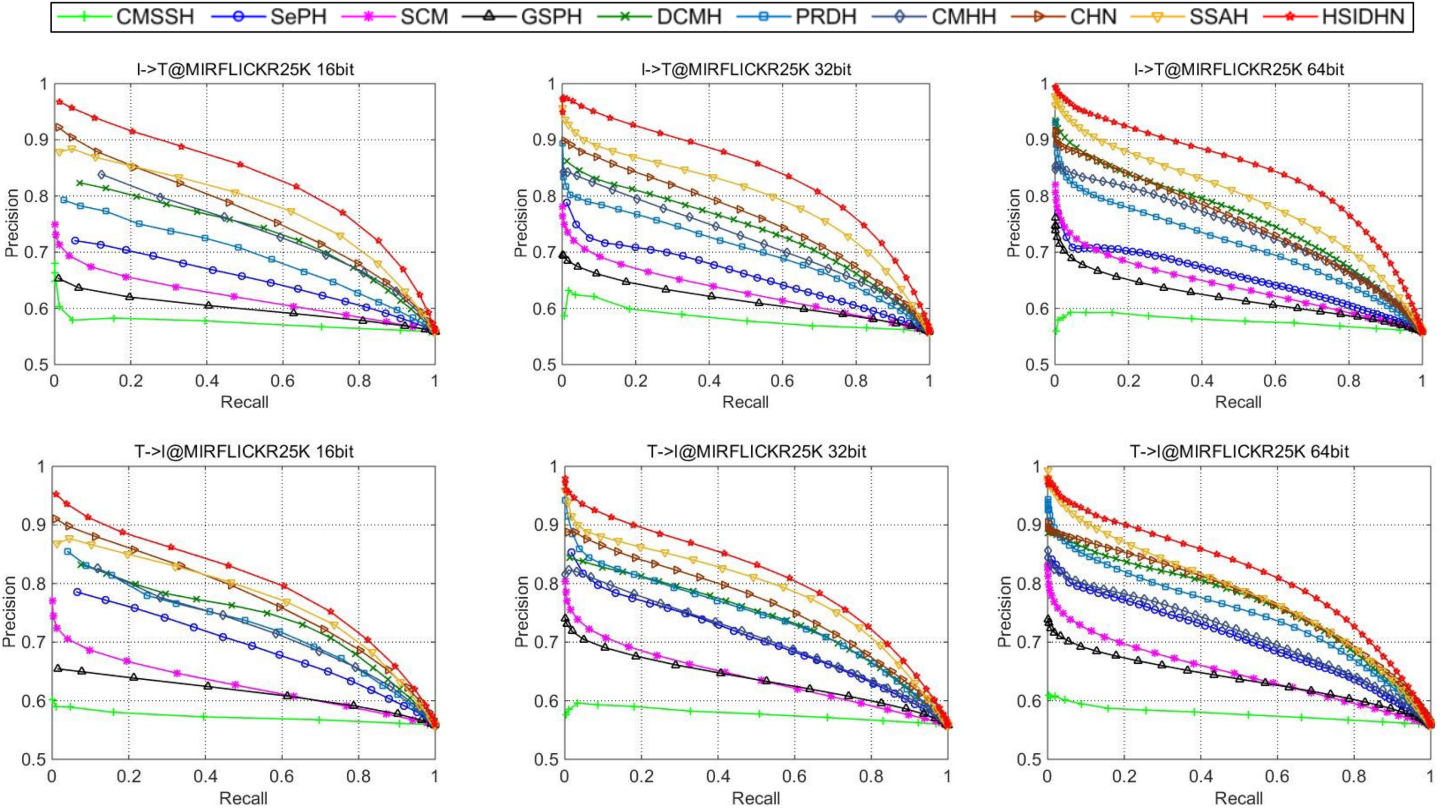
Performance on MIRFlickr-25K evaluated by PR curves.

**Figure 4:**
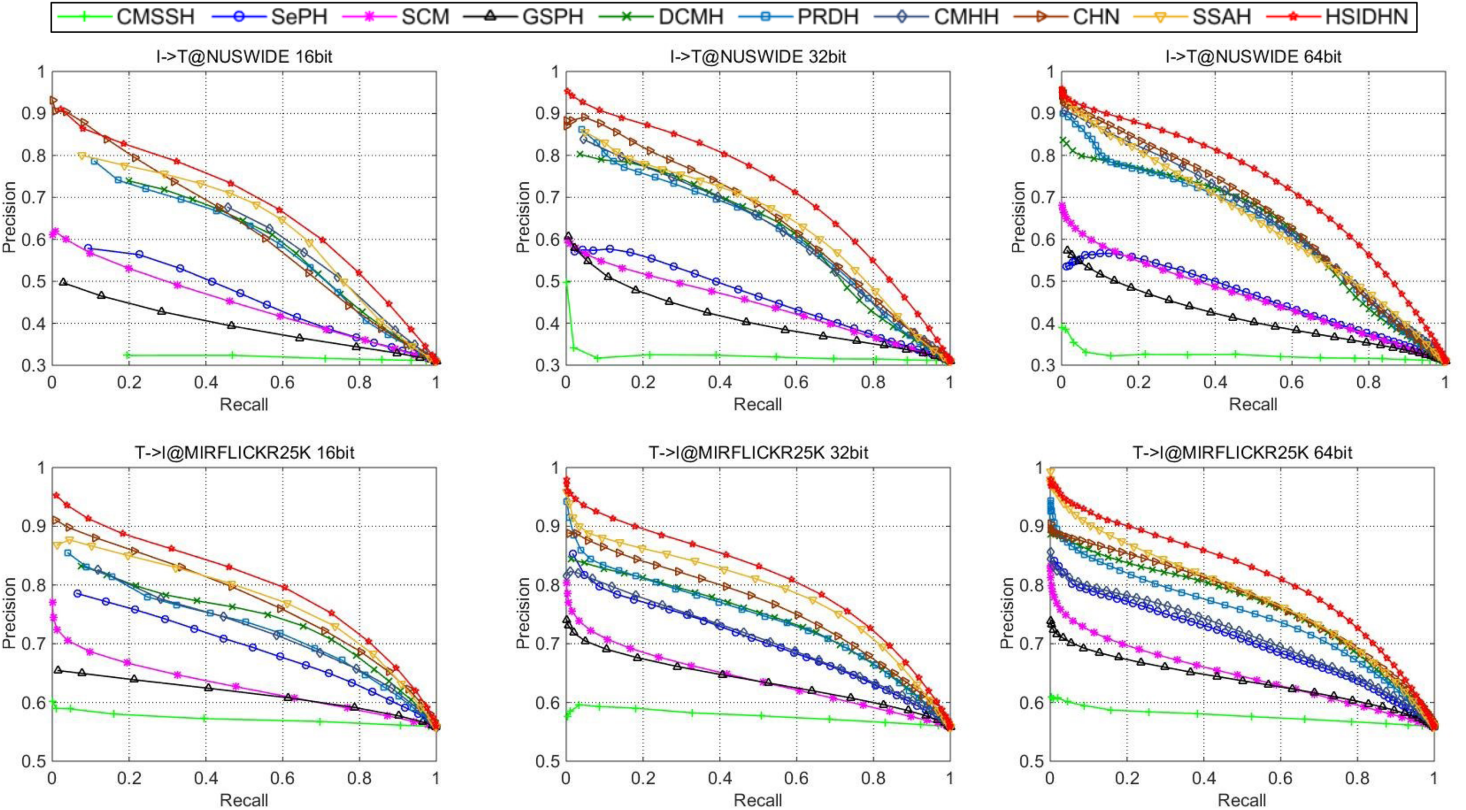
Performance on NUS-WIDE evaluated by PR curves.

The HSIDHN is compared with several baseline methods including SCM (Zhang & Li, 2014), DCMH (Jiang & Li, 2017), CMHH (Cao et al., 2018), PRDH (Yang et al., 2017), CHN (Cao et al., 2016), SepH (Lin et al., 2015) and SSAH (Li et al., 2018). Table 2 and Table 3 illustrates the MAP results of HSIDHN and other methods in different lengths of hash codes. Fig. 3 and Fig. 4 demonstrate the PR curves of different length of hash codes conducted on MIRFlickr-25K and NUS-WIDE. From the result, we can get the following observations and analysis.

- HSIDHN dramatically exceeds other methods on different lengths of hash codes in consideration of the MAP, which reveals the advantages of the multi-scale and multi-level interaction module. It is worth nothing that HSIDHN outperform DCMH by 9.8–3.9% and 17.77–12.93% in terms of MAP for Image-query-Text and Text-query-Image tasks on MIRFLICKR-25K and NUS-WIDE. This is mainly because that the multi-scale process could explore different receptive field of input data, where information with different size could be fully used. Additionally, the hierarchical feature interaction could explore the useful specific feature from different layer and integrated them to enhance the capability of final hash representations.
- The high performance of HSIDHN is partly because the semantic relation and correlation from different intermediate layers are explored by bidirectional bi-linear interaction module. Besides, the multi-scale fusion could further make full use of leverage spatial information.
- There is a kind of imbalance between the performance of image-query-text and text-query-image in almost all the other baselines. However, this phenomenon could be effectively avoided in HSIDHN. This is mainly due to the dual-similarity measurement, which can be sufficient to unify the image-modality and text-modality in the latent common space.
- All deep CMH methods, including DCMH, CHN, PRDH, CMHHH, and SSAH obtain higher performance than other shadow hashing methods such as SePH and SCM. This demonstrates the effectiveness and efficiency of deep neural networks in hash representations and hash function learning, which is more robust than the non-deep neural network methods. Thus, deep neural network based deep hashing methods could obtain better performance.

**Table 2:** Mean Average Percision (MAP) comparison results for MIRFlickr-25K.

	MIRFLICKR-25K					
Method	Image-query-text			Text-query-image		
	16 bits	32 bits	64 bits	16 bits	32 bits	64 bits
SCM Zhang & Li (2014)	0.6354	0.5618	0.5634	0.6340	0.6458	0.6541
SePH Lin et al. (2015)	0.6740	0.6813	0.6830	0.7139	0.7258	0.7294
DCMH Jiang & Li (2017)	0.7316	0.7343	0.7446	0.7607	0.7737	0.7805
CHN Cao et al. (2016)	0.7504	0.7495	0.7461	0.7776	0.7775	0.7798
PRDH Yang et al. (2017)	0.6952	0.7072	0.7108	0.7626	0.7718	0.7755
SSAH Li et al. (2018)	0.7745	0.7882	0.7990	0.7860	0.7974	0.7910
CMHH Cao et al. (2018)	0.7334	0.7281	0.7444	0.7320	0.7183	0.7279
HSIDHN	0.7978	0.8097	0.8179	0.7802	0.7946	0.8115

**Table 3:** Mean Average Percision (MAP) comparison results for NUS-WIDE.

	NUS-WIDE					
Method	Image-query-text			Text-query-image		
	16 bits	32 bits	64 bits	16 bits	32 bits	64 bits
SCM Zhang & Li (2014)	0.3121	0.3111	0.3121	0.4261	0.4372	0.4478
SePH Lin et al. (2015)	0.4797	0.4859	0.4906	0.6072	0.6280	0.6291
DCMH Jiang & Li (2017)	0.5445	0.5597	0.5803	0.5793	0.5922	0.6014
CHN Cao et al. (2016)	0.5754	0.5966	0.6015	0.5816	0.5967	0.5992
PRDH Yang et al. (2017)	0.5919	0.6059	0.6116	0.6155	0.6286	0.6349
SSAH Li et al. (2018)	0.6163	0.6278	0.6140	0.6204	0.6251	0.6215
CMHH Cao et al. (2018)	0.5530	0.5698	0.5924	0.5739	0.5786	0.5889
HSIDHN	0.6498	0.6787	0.6834	0.6396	0.6529	0.6792

### Ablation study

In this section, the importance of each component of HSIDHN is validated. To evaluate the effect of the different modules, the settings of experimental are defined as:

- HSIDHN-SIM is designed by replacing the dual-similarity by single hamming distance measurement.
- HSIDHN-BBI is designed by removing the interaction between layers, and the final hash representations are generated from the final layer of the network.

The results of the ablation study are shown in Table 4. Firstly, there is no doubt that the dual-similarity measurement is better than the hamming-based distance. This is mainly due to that the fine-grained dual-similarity could better preserve the semantic relationship. Moreover, the performance experiences a significant drop when the BBI policy is removed. This may partly because the BBI policy can explore the more robust hash representations from intermediate layers of networks.

**Table 4:** Ablation study results.

Method	MIRFLICKR-25K		NUS-WIDE	
	Image-query-text	Text-query-image	Image-query-text	Text-query-image
HSIDHN-SIM	0.8140	0.8097	0.6432	0.6401
HSIDHN-BBI	0.8034	0.8004	0.6316	0.6275
HSIDHN	0.8179	0.8115	0.6834	0.6792

### Time complexity

The Eq. (31) is taken as the final loss function to train. Each term of the Eq. (31) is MSE loss or max log-likelihood loss which are general in cross-modal retrieval applications. A server with a Titan Xp card is leveraged to train. For the whole HSIDHN, the training and validation procedure need around 28 h for MIRFLICKR-25K and 53 h for NUS-WIDE. The proposed HSIDHN have a fast convergence rate than other deep hashing methods, as the introduction of bidirectional bi-linear interaction and dual-similarity measurement.

### Limitation of HSIDHN and future work

Although the appealing performance has been obtained in the HSIDHN framework, there are still some limitations. Firstly, the network architecture, especially the multi-scale and multi-level features extraction process, requires huge GPU memory to train. The model compression might be the possible solution to solve it. Secondly, the performance of text-query-image is not as significant as image-query-text. This is partly because of the sparsity of features learning from text modality. Some pretraining model is the possible way to learn higher quality features from the original text.

## Conclusion

In this paper, an efficient and effective framework called HSIDHN is proposed for cross-modal hashing retrieval tasks. HSIDHN has three main benefits over the existing methods in CMH community. Firstly, a multi-scale fusion and a Bidirectional Bi-linear Interaction (BBI) module are designed in our framework, with the goal of learning modal-specific hash representations and discriminative hashing codes. Additionally, a dual-similarity measurement strategy is proposed to calculate the fine-grained semantic similarity for both intra and inter-modality pairwise labels. Finally, but certainly not least, experimental results on two large scale benchmark datasets illustrate the superiority of HSIDHN compared with other baseline methods.

## Additional Information and Declarations

### Data Availability

The following information was supplied regarding data availability: MIRFLICKR-25K is available at https://press.liacs.nl/mirflickr/#sec_download. Cite as: M. J. Huiskes, M. S. Lew (2008). The MIR Flickr Retrieval Evaluation. ACM International Conference on Multimedia Information Retrieval (MIR'08), Vancouver, Canada. NUS-WIDE is available at https://lms.comp.nus.edu.sg/wp-content/uploads/2019/research/nuswide/NUS-WIDE.html. Cite as: Tat-Seng Chua, Jinhui Tang, Richang Hong, Haojie Li, Zhiping Luo, and Yan-Tao Zheng. "NUS-WIDE: A Real-World Web Image Database from National University of Singapore", ACM International Conference on Image and Video Retrieval. Greece. Jul. 8-10, 2009. The source code is available at GitHub: https://github.com/SuperChanS/DMMIH.
